# Responses of Arbuscular Mycorrhizal Fungi Diversity and Community to 41-Year Rotation Fertilization in Brown Soil Region of Northeast China

**DOI:** 10.3389/fmicb.2021.742651

**Published:** 2021-10-11

**Authors:** Shiyu Zhang, Peiyu Luo, Jinfeng Yang, Muhammad Irfan, Jian Dai, Ning An, Na Li, Xiaori Han

**Affiliations:** ^1^College of Land and Environment, Shenyang Agricultural University, Shenyang, China; ^2^National Engineering Laboratory for Efficient Utilization of Soil and Fertilizer Resources, Shenyang, China; ^3^Scientific Observation and Experiment Station of Corn Nutrition and Fertilization in Northeast Agricultural and Rural Areas, Shenyang, China; ^4^Department of Biotechnology, University of Sargodha, Sargodha, Pakistan

**Keywords:** arbuscular mycorrhizal fungi, AMF community composition, long-term fertilization, Illumina Miseq sequencing, brown soil, maize

## Abstract

Arbuscular mycorrhizal fungi (AMF) play vital roles in the growth and development of plants, ecosystem sustainability, and stability in agroecosystem, such as transporting nutrients to host plants, improving soil physical structure, and enhancing the stress resistance of host plants. However, the effects of fertilization on AMF diversity and community in brown soil areas are still unclear. The purpose of this study is to explore changes in AMF diversity and community structures and finding out the factors that influenced the changes after 41 years of fertilization in brown soil. Samples were collected from five treatments of the long-term fertilization experiment in June 2019, including CK (no fertilizer), N (mineral nitrogen fertilizer), NP (mineral nitrogen and phosphate fertilizer), M (pig manure), and MNP (pig manure, mineral nitrogen, and phosphate fertilizer). Illumina HiSeq sequencing was used to determine AMF diversity and community structure. The relationship between AMF communities in soil and roots and environmental factors was analyzed by redundancy analysis. The results showed that the soil nutrient content of manure treatments was generally higher than that of chemical fertilizer treatments and no fertilizer treatment. Long-term fertilization increased AMF spore density, which increased with the increase of soil fertility. The moderate content of soil available phosphorus was beneficial to the colonization of AMF. AMF diversity in soil decreased with soil fertility, but AMF diversity in roots was influenced only by soil nitrate–nitrogen and pH. Glomus was the dominant genus in both soil and root samples. AMF community structure in soil and roots had a different response to long-term fertilization. Application of manure had a greater impact on AMF community structure in soil, whereas application of exogenous phosphate fertilizer had a greater impact on that in roots. Soil ammonium nitrogen, nitrate–nitrogen, total nitrogen, organic carbon, total potassium, and available potassium were the most important factors that influenced taxa of AMF in soil, whereas soil ammonium nitrogen, nitrate–nitrogen, total nitrogen, organic carbon, total potassium, available potassium, available phosphorus, and plant phosphorus and potassium content were the most important factors influencing taxa of AMF in maize roots under long-term fertilization in brown soil.

## Introduction

Arbuscular mycorrhizal fungi (AMF) can form an obligate symbiosis with more than 80% of land plants in natural ecosystems (Schüßler et al., 2001). The mycorrhizal plays a variety of important roles such as improving soil physical structure (Rillig, 2004), transporting nutrients to host plants (Hodge and Storer, 2015), promoting plant growth (Higo et al., 2018), enhancing the stress resistance of host plants (Tao et al., 2016), contributing to host plants’ resistance to pathogen invasion (Smith and Read, 2008), and regulating aboveground plant diversity and ecosystem stability. Most crops (95%), such as corn, rice, wheat, potatoes, and soybeans, form symbiotic relationships with AMF (Hijri, 2016). It is gradually recognized that AMF play key roles in the agroecosystem (Lin et al., 2012). Because of the importance of AMF to soil structure and plant growth, many researchers have focused attention on the effects of agricultural management on the AMF community (Gosling et al., 2010). However, some research results were contradictory.

AMF may be sensitive to changes in soil nutrients due to the important symbiotic relationship between mycorrhizal fungi and host plants (Hack et al., 2018). Many previous studies have shown that mineral fertilizer input has (positive, negative, or insignificant) effects on AMF growth by altering the soil microenvironment. A 6-year field experiment in Inner Mongolia showed that N application mainly changed the species composition of AMF, whereas P application affected the abundance of AMF (Chen et al., 2014). However, Xiao et al. (2019) revealed that the addition of N affected the abundance of AMF, whereas the addition of P affected the diversity of AMF, and the addition of N and P had no significant effect on the community composition of AMF in the karst ecosystem. Other studies have shown that high soil nutrient content such as N and P can promote AMF sporulation, the input of organic fertilizer is beneficial to the growth of soil flora, and soil pH and K have significant effects on the community composition of AMF (Qin et al., 2015). It has been shown that some AMF species exist only in acidic or alkaline soils, whereas others can exist in both (Xiao et al., 2019). A study in southern Sweden revealed that different agricultural practices, especially conventional management, had significant effects on AMF community composition and reduced AMF diversity. Organic agriculture could maintain a greater AMF diversity than conventional agriculture (Manoharan et al., 2017). It has also been shown that traditional farming generally reduces the abundance of AMF compared to no-tillage (Gu et al., 2020), mainly due to the destruction of the mycelial network of AMF (Jansa et al., 2006). In addition, host plants also affect the soil AMF community. Jiang et al. (2020) revealed that the relative abundance of *Glomus* and *Scutellospora* in mango orchards was significantly higher than that in litchi orchards, whereas the relative abundance of *Diversispora*, *Acaulospora*, *Ambispora*, and *Paraglomus* in litchi orchards were significantly higher. AMF abundance in July was higher than that in December, whereas the results of richness and Chao1 index were opposite (Xiao et al., 2019). In addition, much of the previous researches has been based on short-term responses, which can be quite different from long-term responses to different fertilizers. In general, long-term fertilization has a greater effect on soil properties and microbial communities. In this light, AMF may be influenced by various factors, such as fertilization method, tillage system, soil type, host plant, and sampling time (Barnes et al., 2016; Gu et al., 2020; Jiang et al., 2020). Most of the previous studies focused on the effects of fertilization on AMF in soil, and there were few studies on the effects of fertilization on AMF in field crop roots. Therefore, a more systematic study on the response of AMF communities in soil and roots to long-term fertilization can better explore the potential functions of AMF species and communities. However, although the effects of different agricultural practices on AMF have been studied in different areas, it had not been determined in brown soil areas. Therefore, this study aimed to investigate the AMF community structure and diversity in soil and roots under long-term different fertilization and to explore the relationship between AMF diversity and community structure and soil characteristics.

In this study, high-throughput sequencing was used to analyze AMF diversity and community composition of soil and maize roots under long-term fertilization in brown soil. Our objectives were to clarify the following two issues: (i) whether long-term fertilization changed the root colonization rate and community structure of AMF and (ii) the most important factors affecting the community composition of AMF in brown soil. The results of this study are expected to provide a basis for the future utilization of indigenous AMF.

## Materials and Methods

### Experimental Description

The long-term fertilization experiment has been conducted since 1979 in Shenyang, Liaoning Province, northeastern China (40°48′N, 123°33′E). This region is a temperate subhumid continental climate with an average annual temperature of 7.0–8.1°C. The average annual precipitation is approximately 574 to 684 mm. The details of the experimental soil were described by Luo et al. (2015). The initial field soil (0–20 cm) contained 15.90 g/kg organic matter, 0.80 g/kg total nitrogen (TN), 0.38 g/kg total phosphorus (TP), 21.10 g/kg total potassium (TK), 105.5 mg/kg alkali-hydrolyzable nitrogen, 6.50 mg/kg available phosphorus (AP), and 97.90 mg/kg available potassium (AK) and had a pH of 6.50 in 1979. The experimental field was arranged in a randomized block design under a rotation of maize–maize–soybean. Detailed information was described by Li et al. (2020). In this study, we selected five treatments including CK (no fertilizer), N (mineral nitrogen fertilizer), NP (mineral nitrogen and phosphate fertilizer), M (pig manure), and MNP (pig manure, mineral nitrogen, and phosphate fertilizer). The chemical fertilizers were applied in the form of urea, calcium superphosphate, and potassium sulfate, respectively. The average nutrient content of pig manure in 41 years was as follows: 83.5 g/kg organic C, 7.2 g/kg N, 3.8 g/kg P, and 8.3 g/kg K. Fertilizer application rates of these treatments are detailed in Supplementary Table 1. The field was plowed (20-cm depth) before planting. The selected maize variety was *Zea mays L.* “Dongdan6531.” It was sown on May 4 and harvested on September 28, 2019. The planting density of maize is 60,000 plants ha^–1^.

### Sample Collection and DNA Extraction

Soil and root samples were collected in June 2019 (maize seedling stage). Twelve soil samples (0- to 20-cm depth) were randomly collected with a soil drilling sampler (diameter 5 cm) from each plot and thoroughly mixed as one composite sample. Fresh soil samples were removed from the visible plant residues, roots, stones, and through a sieve (<2 mm). The sieved samples were divided into two parts. One subsample was used to determine soil ammonium nitrogen (NH_4_^+^-N), soil nitrate nitrogen (NO_3_^–^-N), and soil dissolved organic carbon (DOC) and to extract soil DNA; the other subsample was air-dried for determination of soil basic physicochemical properties and AMF spore density. Four complete maize roots were collected from each plot, rinsed with tap water, and then rinsed with sterilized ultrapure water. The fresh roots were cut into 1-cm segments and fully mixed for DNA extraction and quantification of mycorrhizal colonization. Six plants were collected from each plot and inactivated the enzyme activity at 105°C for 30 min and then were oven-dried at 65°C to constant weight and finally ground.

Total DNA was extracted from soil using the Powersoil^®^ DNA isolation kit (Mo Bio Laboratories, Inc., Carlsbad, CA, United States) according to the manufacturer’s instructions. Root samples were milled with liquid nitrogen, and DNA was extracted using the DNeasy^®^ Plant Mini Kit (Qiagen, Hilden, Germany). The content and quality of extracted DNA were examined using a NanoDrop ND-1000 (NanoDrop Technologies Inc., Wilmington, DE, United States) and then stored at −20°C until further analyses.

### Spore Density and Root Colonization of Arbuscular Mycorrhizal Fungi

Arbuscular mycorrhizal fungi spores were extracted and counted using Daniels and Skipper (1982) method. A 0.5-g root sample was cleared with 10% (wt/vol) KOH at 90°C for 2 h and stained with trypan blue, and the AMF root colonization was quantified by Giovannetti and Mosse (1980) method.

### Soil Property and Plant Nutrient Content Measurements

Soil NH_4_^+^-N and NO_3_^–^-N were extracted with 1 M KCl solution and then measured using an autoanalyzer (AutoAnalyzer3, SEAL Analytical, Germany). TN and total carbon were determined with an Element Auto-Analyzer (Vario MAX CN; Elementar, Hanau, Germany). TP and AP were measured according to Murphy and Riley (1962) method. TK and AK was measured according to Schollenberger and Simon (1945) method. DOC was extracted using the method of Jones and Willett (2006). DOC concentration was determined by a total organic carbon analyzer (Vario EL II, Germany). Soil pH was measured using a pH meter (Metter-Toledo 320) in a soil and water mixture (wt/vol = 1:2.5). The plant samples were digested in a mixture of concentrated H_2_SO_4_ and H_2_O_2_. Digests were analyzed for the content of nitrogen in plants by the Automatic Kjeldahl Apparatus (K9840, Hanon, Shandong, Hanon Ltd., China), for the content of phosphorus in plants by the vanadium molybdate yellow colorimetric method (Delaporte-Quintana et al., 2017), and the content of potassium in plants was measured by the flame photometer (M410, Sherwood Scientific Ltd., United Kingdom).

### Polymerase Chain Reaction Amplification

AMF DNA was amplified using a nested polymerase chain reaction (PCR) with a first primer pair of LR1 (5′-GCATATC AATAAGCGGAGGA-3′) and FLR2 (5′-GTCGTTTAAAG CCATTACGTC-3′) (Van Tuinen et al., 1998) and a second primer pair of FLR3 (5′-TTGAAAGGGAAACGATTGAAGT-3′) and FLR4 (5′-TACGTCAACATCCTTAACGAA-3′) (Gollotte et al., 2004). The 25-μL reaction system in both rounds of PCR contained 12.5 μL 2 × Taq PCR MasterMix, 1 μL of each primer pair (5 μM), 3 μL of bovine serum albumin (2 ng/μL), and template DNA (30 ng of DNA from soil and root for the first PCR and 1 μL of product from the first PCR for the second PCR) and adjusted with sterile double distilled water to a final volume of 25 μL. For amplification, the PCR thermal cycle conditions were an initial denaturation step at 98°C for 30 s and 30 cycles of denaturation at 95°C for 5 s, annealing at 58°C for 6 s, and extension at 72°C for 10 s, followed by a final extension at 72°C for 90 s (two rounds of PCR had the same thermal cycle conditions). The samples were sequenced on MiSeq platform at Allwegene Company, China.

### Illumina HiSeq Sequencing and Bioinformatics Analyses

The raw data were screened and removed according to the standard protocols by Allwegene Technology Inc. (Beijing, China). Qualified reads were separated and trimmed with Illumina Analysis Pipeline version 2.6. The dataset was analyzed using search (version 8.1). The sequences were clustered into operational taxonomic units (OTUs) at a similarity level of 97%. The RDP Classifier tool was used to classify all sequences into different taxonomic groups. The raw sequence data had been accessioned in the NCBI Sequence Read Archive database (accession no. PRJNA749871).

### Statistical Analyses

The measured data were analyzed using SPSS 21.0 (SPSS Inc., Chicago, IL, United States). The analysis of differences between treatments was performed using one-way analysis of variance (ANOVA) followed by Duncan test. The relationships between soil physicochemical properties and AMF parameters were analyzed using the Spearman correlation. The non-metric multidimensional scaling (NMDS) was conducted using the “vegan” package for R software (v.3.1.0) based on the Bray–Curtis distance of OTUs. Linear discriminant analysis (LDA) was used to estimate the significant differences among AMF groups under different fertilization treatments. Redundancy analyses (RDAs) (Canoco for Windows version 5.0.) were used to analyze the relationship between soil properties, plant nutrient contents, and AMF communities in the soil and roots, and associated *F* values in the RDA were calculated.

## Results

### Soil Physicochemical Properties and Plant Nutrient Content

After 41 years of long-term fertilization, soil physicochemical properties under different fertilization treatments showed obvious differences (Table 1). The contents of NH_4_^+^-N, NO_3_^–^-N, AP, AK, and DOC in the soil were 3.71–7.75, 12.06–73.63, 1.19–188.81, 81.62–288.12, and 39.42–126.99 mg/kg, while the contents of SOC, TN, TP, and TK in the soil were 9.08–16.35, 0.90–1.68, 0.32–1.16, and 15.62–20.58 g/kg in all treatments, respectively. Compared with the CK treatment, long-term fertilization increased the content of NH_4_^+^-N, NO_3_^–^-N, DOC, SOC, and TN, and the highest content of these soil nutrients was in the MNP treatment. The soil pH value ranged from 4.87 to 6.64. Compared with the CK treatment, long-term application of chemical fertilizer significantly reduced the soil pH value, and the lowest soil pH value (4.87) was in the N treatment. Long-term application of chemical fertilizer reduced the pH value by 1.43–1.63 units compared to the initial value (6.50) in 1979. However, the long-term application of manure could significantly alleviate soil acidification, and the soil pH value (6.64) was the highest in the M treatment.

**TABLE 1:** Soil properties for different fertilizer treatments.

Treatment	NH_4_^+^-N	NO_3_^—^N	AP	AK	DOC	SOC	TN	TP	TK	pH
	(mg⋅kg^–1^)	(mg⋅kg^–1^)	(mg⋅kg^–1^)	(mg⋅kg^–1^)	(mg⋅kg^–1^)	(g⋅kg^–1^)	(g⋅kg^–1^)	(g⋅kg^–1^)	(g⋅kg^–1^)	(H_2_O)
CK	3.71 ± 0.10e	12.06 ± 0.03e	1.19 ± 0.11d	96.66 ± 2.09b	39.42 ± 6.24e	9.08 ± 0.08e	0.90 ± 0.00e	0.32 ± 0.02c	15.68 ± 0.64c	5.75 ± 0.01b
N	4.45 ± 0.22d	48.38 ± 1.78b	2.14 ± 0.11d	81.62 ± 3.22c	77.60 ± 1.87c	10.28 ± 0.01d	1.03 ± 0.01d	0.34 ± 0.03c	16.45 ± 0.46c	4.87 ± 0.00d
NP	4.83 ± 0.13c	45.89 ± 1.28c	16.15 ± 0.40c	76.94 ± 4.01c	57.15 ± 5.68d	11.51 ± 0.06c	1.06 ± 0.00c	0.32 ± 0.01c	15.62 ± 0.69c	5.07 ± 0.01c
M	5.33 ± 0.17b	38.47 ± 0.79d	137.72 ± 3.97b	223.30 ± 8.02a	111.06 ± 8.92b	12.87 ± 0.01b	1.33 ± 0.01b	0.66 ± 0.07b	18.02 ± 0.38b	6.64 ± 0.06a
MNP	7.75 ± 0.21a	73.63 ± 0.74a	188.81 ± 8.29a	288.12 ± 6.22a	126.99 ± 1.26a	16.35 ± 0.02a	1.68 ± 0.01a	1.16 ± 0.02a	20.58 ± 0.30a	5.78 ± 0.02b

*Values are means (*n* = 3) ± SD. Statistical significance was set at a level of *p* < 0.05 using Duncan multiple-range tests. The same letter in the table represents no significant difference. NH_4_**^+^**-N, ammonium nitrogen; NO_3_**^–^**-N, nitrate nitrogen; AP, available phosphorus; AK, available potassium; DOC, dissolved organic carbon; SOC, soil organic carbon; TN, total nitrogen; TP, total phosphorus; TK, total potassium; CK, no fertilizer; N, mineral nitrogen fertilizer; NP, mineral nitrogen and phosphate fertilizer; M, pig manure; MNP, pig manure, mineral nitrogen, and phosphate fertilizer.*

The biomass of maize plant and the plant nutrient content also varied among different treatments (Table 2), with the highest nutrient content in the MNP treatment and the lowest nutrient content in the CK treatment. Except for the N treatment, the plant biomass of all fertilization treatments was higher than that of the CK treatment.

**TABLE 2:** Plant nutrient content and biomass.

Treatment	N concentration	P concentration	K concentration	N content	P content	K content	Biomass
	(%)	(%)	(%)	(mg/plant)	(mg/plant)	(mg/plant)	(g/plant)
CK	2.68 ± 0.09b	0.17 ± 0.02c	3.15 ± 0.04b	10.63 ± 0.34c	0.67 ± 0.08c	14.31 ± 3.10c	0.40 ± 0.01c
N	3.40 ± 0.47a	0.26 ± 0.03c	3.06 ± 0.11b	16.23 ± 2.21c	1.25 ± 0.12c	14.59 ± 0.50c	0.48 ± 0.01c
NP	3.67 ± 0.17a	0.41 ± 0.01b	1.94 ± 0.12c	100.85 ± 4.53b	11.17 ± 0.19b	53.17 ± 3.39b	2.75 ± 0.10b
M	3.69 ± 0.09a	0.63 ± 0.09a	5.67 ± 0.16a	255.18 ± 6.21a	43.36 ± 6.01a	391.63 ± 11.02a	6.90 ± 0.19a
MNP	3.71 ± 0.02a	0.66 ± 0.09a	5.78 ± 0.14a	255.86 ± 1.62a	45.55 ± 6.25a	398.56 ± 9.72a	6.90 ± 0.10a

*Values are means (*n* = 3) ± SD. Statistical significance was set at a level of *p* < 0.05 using Duncan multiple-range tests. The same letter in table represents no significant difference.*

### Spore Density and Root Colonization of Arbuscular Mycorrhizal Fungi

**FIGURE 1:**
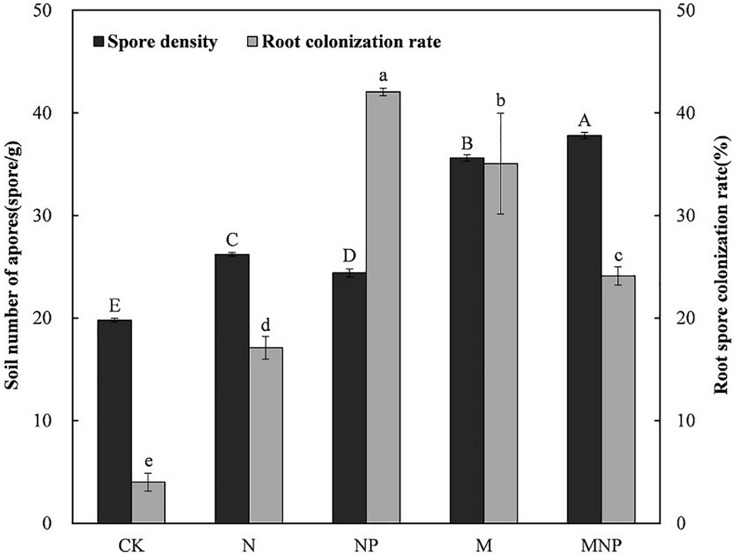
Arbuscular mycorrhizal fungi (AMF) spore density and root colonization rate under different treatments (standard error displayed for *n* = 3). Columns of the same type designated the same letter are not significantly different (*p* < 0.05). CK, no fertilizer; N, mineral nitrogen fertilizer; NP, mineral nitrogen and phosphate fertilizer; M, pig manure; MNP, pig manure, mineral nitrogen, and phosphate fertilizer.

Spore density and root colonization of AMF were significantly influenced by different long-term fertilization (Figure 1). AMF spore density of manure treatments (M, MNP) was significantly higher than that of chemical fertilizer treatments (N, NP) and no fertilizer treatment (CK). AMF spore density of the MNP treatment was the highest (37.8 spores⋅g^–1^ soil), whereas that of the CK treatment was the lowest (19.8 spores⋅g^–1^ soil). Correlation analysis (Supplementary Table 2) showed that AMF spore density was positively correlated with soil nutrient content (*p* < 0.05).

Compared with the CK treatment, long-term fertilization significantly enhanced the colonization of AMF. The colonization rate of AMF to maize roots ranged from 4.02 to 42.03%. The highest colonization rate of AMF was in the NP treatment (42.03%), followed by the M treatment (35.05%). Except for the CK treatment (4.02%), the N treatment had the lowest colonization rate (17.10%). Correlation analysis (Supplementary Table 2) showed that root colonization rate did no correlate with soil physicochemical properties (*P* > 0.05).

### MiSeq Sequencing and Identification of Arbuscular Mycorrhizal Fungi

A total of 481,325 non-chimeric reads from soil samples were classified into 110 OTUs based on a 97% sequence similarity. Among these 110 AMF OTUs, 55 belonged to *Glomeraceae*, 14 to *Claroideoglomeraceae*, 9 to *Paraglomeraceae*, 8 to *Gigasporaceae*, 3 to *Diversisporaceae*, 2 to *Archaeosporaceae*, 1 to *Acaulosporaceae*, and 18 to unidentified OTUs. A total of 566,169 non-chimeric reads from root samples were classified to 125 OTUs based on a 97% sequence similarity. Among these 125 AMF OTUs, 68 belonged to *Glomeraceae*, 15 to *Claroideoglomeraceae*, 17 to *Gigasporaceae*, 7 to *Paraglomeraceae*, 4 to *Diversisporaceae*, and 14 to unidentified OTUs.

To determine whether the sequencing depth of our samples was sufficient to represent AMF diversity, rarefied curves were produced for all samples. The results showed that all rarefaction curves for observed AMF OTUs reached the saturation platform, indicating that the amount of sequencing data was reasonable in this study (Supplementary Figure 1)

### Arbuscular Mycorrhizal Fungi Diversity in Soil and Roots

The Chao1 and observed species index are mainly used to estimate the number of OTUs in the sample. Shannon and Simpson indices reflect the number of species and the uniformity of species abundance in the sample. The AMF abundance and diversity in soil and roots are shown in Table 3. In this study, long-term fertilization changed the alpha diversity of the soil AMF community. In soil, the Chao1 index was higher in the CK, N, and NP treatments, followed by in the M treatment, whereas it was the lowest in the MNP treatment. The Shannon index was higher in the CK and N, followed by in the NP treatment, and the index in the CK, N, and NP treatments was significantly (*p* < 0.05) higher than in the M and MNP treatments, respectively. The Simpson index was higher in the CK and N treatments, followed by the NP and M treatments, and the index was the lowest in the MNP treatment. In roots, the Chao1 index was the highest in the NP treatment, whereas there was no difference in the index among all treatments. The Shannon index was higher in the CK, NP, and M treatments, followed by the MNP treatment, and the index was the lowest in the N treatment. The variation trend of the Simpson index was consistent with the Shannon index. Correlation analysis (Supplementary Table 2) showed that the soil Shannon index was significantly negatively correlated with soil NH_4_^+^-N, AP, SOC, TN, TP, and TK (*p* < 0.01); the root Shannon index had positive correlation with pH (*r* = 0.636, *P* = 0.048) and significant negative correlation with NO_3_^–^-N (*r* = −0.711, *P* = 0.021).

**TABLE 3:** Arbuscular mycorrhizal fungi (AMF) alpha-diversity indices in soil and roots for different fertilizer treatments.

Treatment	Soil				Roots			
	Species richness indices		Species diversity indices		Species richness indices		Species diversity indices	
	Chao1	Observed_species	Shannon	Simpson	Chao1	Observed_species	Shannon	Simpson
CK	89.66 ± 7.61a	78.67 ± 2.31a	4.00 ± 0.07a	0.90 ± 0.00a	106.81 ± 9.62a	96.00 ± 2.00*ab*	4.30 ± 0.12a	0.92 ± 0.01a
N	86.82 ± 5.83a	81.33 ± 1.53a	3.65 ± 0.13a	0.85 ± 0.02a	103.40 ± 14.11a	89.00 ± 2.65*bc*	3.72 ± 0.06c	0.87 ± 0.00c
NP	90.17 ± 3.25a	77.33 ± 1.53a	3.20 ± 0.26b	0.76 ± 0.05b	108.00 ± 12.39a	99.33 ± 4.51a	4.19 ± 0.01a	0.91 ± 0.01a
M	65.68 ± 6.57b	58.33 ± 1.53b	2.59 ± 0.25c	0.75 ± 0.04b	103.95 ± 8.80a	89.67 ± 3.79*b*c	4.29 ± 0.02a	0.92 ± 0.00a
MNP	53.00 ± 6.38c	47.33 ± 9.45c	1.50 ± 0.36d	0.49 ± 0.06c	92.88 ± 6.73a	85.67 ± 6.03c	4.00 ± 0.15b	0.88 ± 0.01b

*Values are means (*n* = 3) ± SD. Statistical significance was set at a level of *p* < 0.05 using Duncan multiple-range tests. The same letter in the table represents no significant difference.*

### Arbuscular Mycorrhizal Fungi Community Composition in Soil and Roots

**FIGURE 2:**
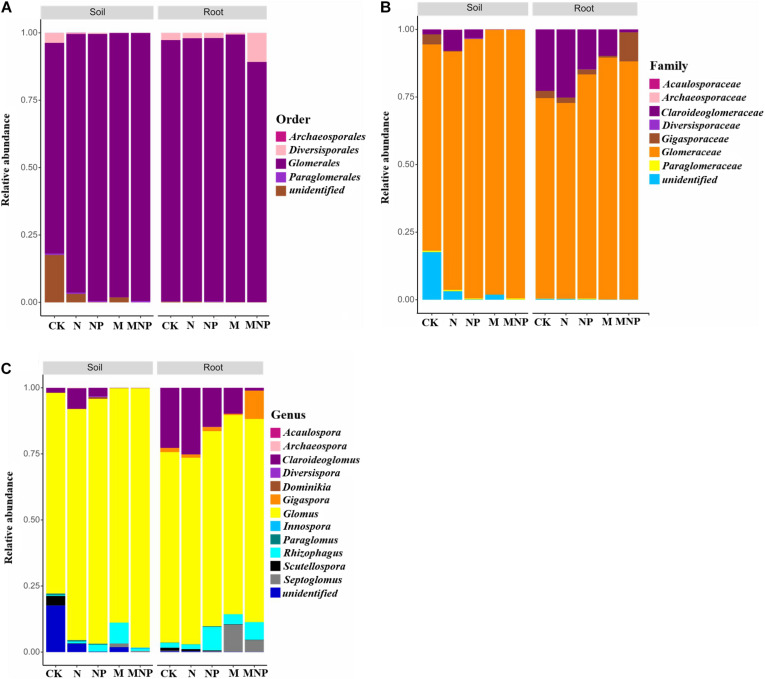
Relative abundance of AMF communities at **(A)** order, **(B)** family, and **(C)** genus level in soil and maize roots under different treatments. CK, no fertilizer; N, mineral nitrogen fertilizer; NP, mineral nitrogen and phosphate fertilizer; M, pig manure; MNP, pig manure, mineral nitrogen, and phosphate fertilizer.

**FIGURE 3:**
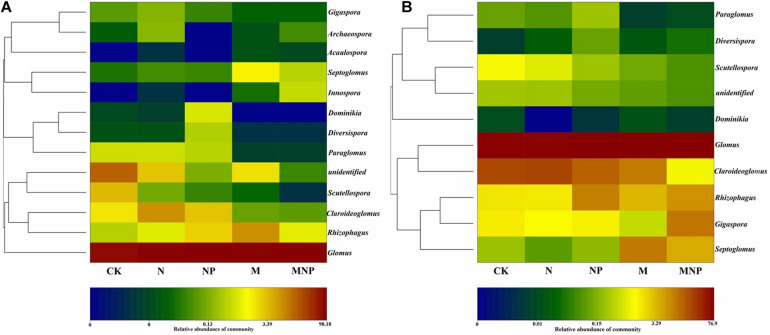
Log-scaled percentage heatmap of AMF genus-level composition in soil **(A)** and roots **(B)**.

AMF communities in soil and roots at order, family, and genus levels are shown in Figure 2. AMF communities belonged to *Glomerales* (78.13–99.42%), *Diversisporales* (0.02–3.71%), *Paraglomerales* (0.02–0.53%), and *Archaeosporales* (0.00–0.17%) in the soil (Figure 2A). AMF communities could be grouped into seven families, including *Glomeraceae*, *Claroideoglomeraceae*, *Gigasporaceae*, *Paraglomeraceae*, *Archaeosporaceae*, *Diversisporaceae*, and *Acaulosporaceae* (Figure 2B). AMF communities could be further grouped into 12 genera, including *Glomus*, *Septoglomus*, *Scutellospora*, *Rhizophagus*, *Claroideoglomus*, *Diversispora*, *Paraglomus*, *Gigaspora*, *Acaulospora*, *Innospora*, *Dominikia*, and *Archaeospora* (Figure 2C). In soil, *Glomus* was the dominant AMF genus for all treatments. Moreover, the relative abundance of *Glomus* in soil was increased by 15.08, 22.03, 16.87, and 29.31% in the N, NP, M, and MNP treatments than in the CK treatment, respectively. Additionally, the relative abundance of *Claroideoglomus*, *Gigaspora*, *Scutellospora*, and *Paraglomus* in soil decreased with the application of organic fertilizers. On the contrary, the relative abundance of *Septoglomus*, *Acaulospora*, and *Innospora* increased with the application of organic fertilizers. Furthermore, the relative abundance of *Scutellospora* was higher in the CK treatment than in other fertilization treatments (Figure 3A). The other genera, including *Paraglomus*, *Dominikia*, *Gigaspora*, *Diversispora*, *Acaulospora*, *Archaeospora*, and *Innospora*, were also found in soil with low relative abundance <1%. The genera *Acaulospora*, *Archaeospora*, and *Innospora* were found only in soil and not detected in the maize root samples.

AMF communities belonged to *Glomerales*, *Diversisporales*, and *Paraglomerales* were found in maize root samples (Figure 2). The most abundant order was *Glomerales*; its abundance was higher in M treatment (99.27%) and lower in MNP (89.12%). AMF communities could be grouped into five main families, including *Glomeraceae*, *Claroideoglomeraceae*, *Gigasporaceae*, *Paraglomeraceae*, and *Diversisporaceae*, and the most abundant family was Glomeraceae. AMF communities could be further grouped into nine genera, including *Glomus*, *Claroideoglomus*, *Gigaspora*, *Paraglomus*, *Septoglomus*, *Scutellospora*, *Diversispora*, *Rhizophagus*, and *Dominikia*. *Glomus* and *Claroideoglomus* were the dominant AMF genera in roots at all treatments. Moreover, the variation trend of the relative abundance of *Claroideoglomus*, *Scutellospora*, *Paraglomus*, and *Septoglomus* in root samples was similar to those in soil (Figure 3B). *Diversisporales*, *Paraglomus*, and *Dominikia* were also found in maize root samples with low relative abundance <1%.

**FIGURE 4:**
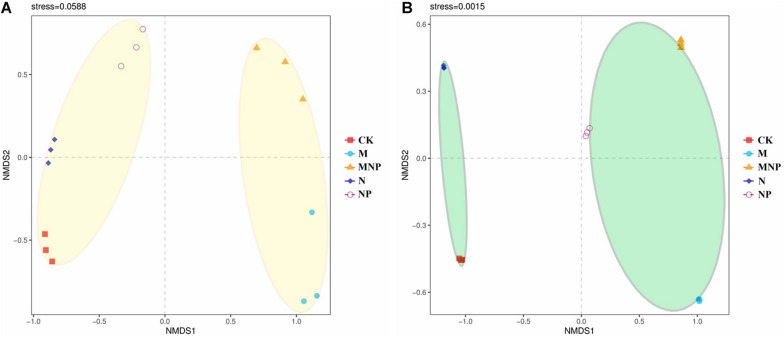
Non-metric multidimensional scaling (NMDS) of AMF community composition in soil **(A)** and roots **(B)**.

The community composition of AMF was analyzed using NMDS based on Bray–Curtis similarity distance (Figure 4). AMF communities in soil were divided into two groups; organic fertilizer treatments were clustered together into one group, whereas no organic fertilizer treatments were clustered together into the other group (Figure 4A). AMF communities in roots were divided into two groups; adding exogenous phosphate fertilizer treatments were clustered together into the one group, and no-adding exogenous phosphate fertilizer treatments were clustered together into the other group (Figure 4B).

**FIGURE 5:**
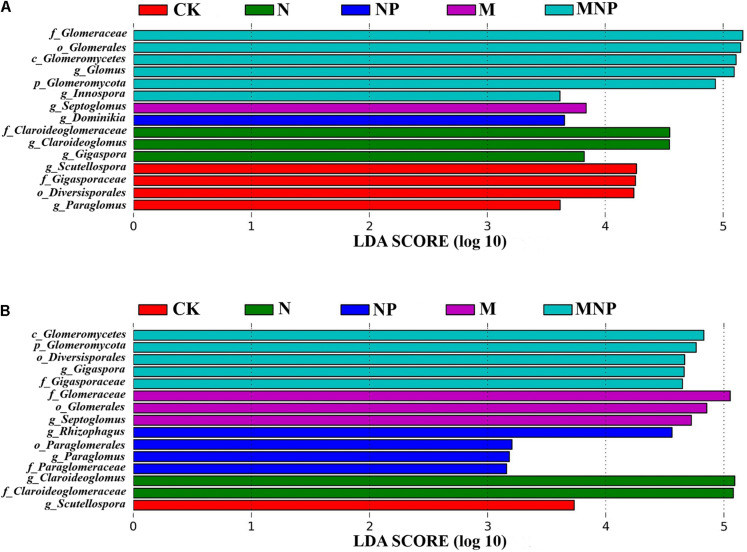
The LDA (linear discriminant analysis) distribution histogram of biomarkers in the soil **(A)** and roots **(B)** under different fertilization treatments. Only taxa with LDA values greater than 3.0 (*p* < 0.05) are shown.

The LDA was used to show the significant difference of AMF species in soil and maize roots under different fertilization treatments (Figure 5). Biomarkers with a statistically significant LDA score greater than 3 were demonstrated. At the genus level, the relative abundance of *Glomus* and *Innospora* in the soil was significantly higher in the MNP treatment, *Septoglomus* was significantly higher in the M treatment, *Dominikia* was significantly higher in the NP treatment, *Claroideoglomus* and *Gigaspora* were significantly higher in the N treatment, and *Scutellospora* and *Paraglomus* were significantly higher in the CK treatment (Figure 5A). The relative abundance of *Gigaspora* in maize roots was significantly higher in the MNP treatment, *Septoglomus* was significantly higher in the M treatment, and *Rhizophagus* and *Paraglomus* were significantly higher in the NP treatment, whereas *Claroideoglomus* was significantly higher in the N treatment, and *Scutellospora* was significantly higher in the CK treatment (Figure 5B).

### Factors Affecting Arbuscular Mycorrhizal Fungi Community

**FIGURE 6:**
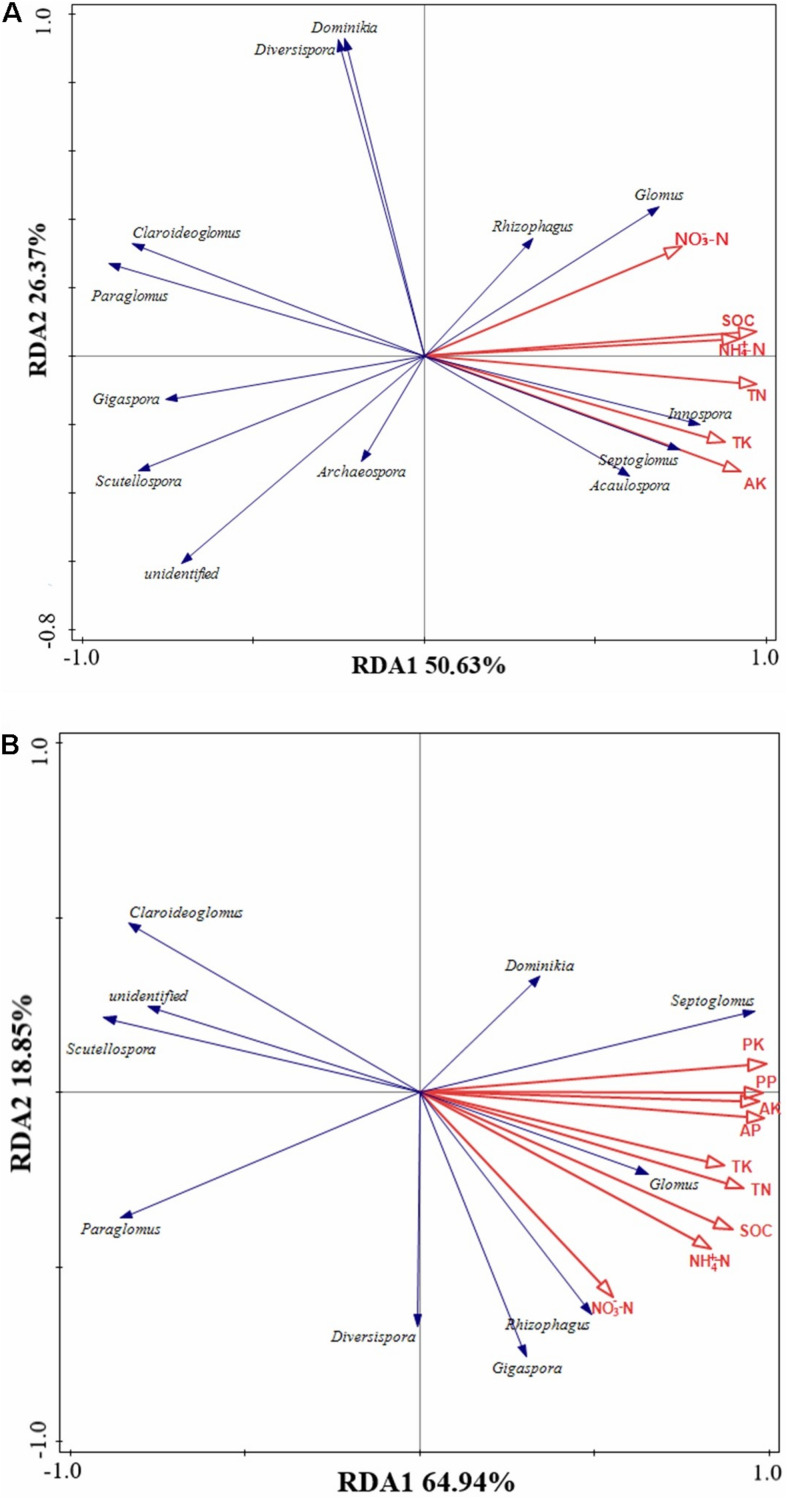
Redundancy analysis (RDA) of AMF communities in soils **(A)** and roots **(B)** in response to different fertilizer applications. NH_4_^+^-N, ammonium nitrogen; NO_3_^–^-N, nitrate nitrogen; AP, available phosphorus; AK, available potassium; SOC, soil organic carbon; TN, total nitrogen; TP, total phosphorus; TK, total potassium; PP, plant phosphorus content; PK, plant potassium content.

We hypothesized that the effects of long-term fertilization on AMF communities in soil and roots might primarily be mediated by soil physicochemical properties and plant nutrient content. RDA revealed that AMF communities in soil and roots had different responses to environmental factors. The first two ordination axes of RDA explained 77% of the total variation in the AMF community structure of soil (Figure 6A). The most important factors were TN (*F* = 12.3, *p* < 0.01), SOC (*F* = 11.1, *p* < 0.01), NH_4_^+^-N (*F* = 5.1, *p* < 0.01), and NO_3_^–^-N (*F* = 4.9, *p* < 0.01). *Glomus* had a significant positive correlation with NO_3_^–^-N, SOC, NH_4_^+^-N, and TN (*p* < 0.05) in soil. *Acaulospora*, *Septoglomus*, and *Innospora* had a significant positive correlation with SOC, NH_4_^+^-N, TN, TK, and AK (*p* < 0.05) in soil. *Diversispora* and *Dominikia* exhibited a significant negative correlation with AK and TK (*p* < 0.05) in soil. *Claroideoglomus* and *Paraglomus* had a significant negative correlation with SOC, NH_4_^+^-N, TN, TK, and AK (*p* < 0.05) in soil. *Gigaspora* and *Scutellospora* had a significant negative correlation with NO_3_^–^-N, SOC, NH_4_^+^-N, TN, TK, and AK (*p* < 0.05) in soil. *Rhizophagus* and *Archaeospora* in the soil did not correlate with environmental factors.

The first two ordination axes of RDA explained 83.79% of the total variation in the AMF community structure of roots (Figure 6B). The most important factors were plant K content (*F* = 23.3, *p* < 0.01), SOC (*F* = 11.1, *p* < 0.01), AK (*F* = 10.5, *p* < 0.01), and NO_3_^–^-N (*F* = 6, *p* < 0.01). *Glomus* and *Septoglomus* had a significant positive correlation with NH_4_^+^-N, SOC, TN, TK, AP, AK, and plant P and K content (*p* < 0.05) in roots. *Rhizophagus* had a positive correlation with NH_4_^+^-N, SOC, TN, AP, and plant P and K content (*p* < 0.05). *Gigaspora* had a significant positive correlation with NO_3_^–^-N (*p* < 0.05). *Claroideoglomus*, *Scutellospora*, and *Paraglomus* had a significant negative correlation with NH_4_^+^-N, SOC, TN, TK, AP, AK, and plant P and K content (*p* < 0.05). *Diversispora and Dominikia* in roots did not correlate with environmental factors.

## Discussion

### Changes in Soil Properties

Long-term application of chemical fertilizer and manure could significantly increase soil nutrient content, improve soil structure, and further affect soil microorganisms (Lin et al., 2018). In this study, long-term fertilizer significantly increased the content of NH_4_^+^-N, NO_3_^–^-N, DOC, SOC, and TN. However, long-term application of chemical fertilizer can significantly reduce soil pH value, especially the long-term application of N fertilizer alone. The pH value of the soil with long-term application of chemical fertilizer was 1.43–1.63 units lower than that of the initial soil; long-term application of manure could effectively alleviate soil acidification (Table 1). In addition to NO_3_^–^-N, soil nutrients were higher under organic fertilizer treatments (M and MNP). Compared with other fertilization treatments, the content of NO_3_^–^-N was the lowest in the M treatment, which may be related to the relative rate of nutrient release from organic and chemical fertilizers. Our sampling period was the seedling stage of maize, and the rate of nutrient release from pig manure may be slower than that of chemical fertilizer at this time (Wang et al., 2021).

### Arbuscular Mycorrhizal Fungi Spore Density and Root Colonization

Spore density can be affected by many factors, such as host plants, tillage practices, fertilization, and sampling period (Sasvári et al., 2011; Avio et al., 2013; Gu et al., 2020). In this study, the AMF spore density of fertilization treatments was significantly higher than that of no fertilization treatment (Figure 1), which indicated that fertilization had a positive effect on AMF sporulation. The spore density of manure treatments was significantly higher than that of other treatments, indicating that the application of manure was more conducive to AMF sporulation, which was consistent with the results of Qin et al. (2015). The above results might be due to increasing the C: N ratio by adding carbon-enriched pig manure would have favored sporulation. In this study, correlation analysis (Supplementary Table 2) showed that the AMF spore density had a positive correlation with soil nutrients, which confirmed the above hypothesis.

Many previous studies have shown that chemical fertilizers, particularly N and P fertilizers, have negative effects on AMF growth (Bhadalung et al., 2005; Lin et al., 2012), whereas other studies have found no significant effect (Chen et al., 2014; Qin et al., 2015) or positive effects (Hazard et al., 2013). In this study, the root colonization rate of fertilization treatments was significantly higher than that of no fertilization treatment, ranging from 17.10 to 42.03% (Figure 1). Root colonization rate was the highest in the NP treatment, in addition to no fertilizer treatment (CK), the lowest in the N treatment, and manure treatments (M, MNP) was somewhere in between. It might be that soil phosphorus level was moderate in the NP treatment; while applying manure treatment or single N treatment, there was the possibility of soil phosphorus levels being too high or too low. Our speculation has been confirmed by Hack et al. (2018), indicating that the appropriate phosphorus level in the soil is conducive to the colonization of AMF, whereas too high or too low phosphorus level will inhibit the colonization of AMF.

### Arbuscular Mycorrhizal Fungi Diversity in Soil and Roots

Long-term fertilization increased the spore density but decreased soil AMF diversity. This might be that long-term fertilization only increased sporulation of a few AMF; thus, soil diversity decreased rather than increased. In this study, the Shannon index of AMF in soil was significantly lower in manure treatments than in non-manure fertilizer treatments (Table 3), which was due to the decrease or disappearance of some AMF species and the enhancement of some AMF species (Zhang et al., 2021). This result suggested that the higher the soil nutrient content, the less suitable for the growth of some AMF species such as *Scutellospora*, and RDA also confirmed this hypothesis (Figure 6). Studies showed that nitrogen application harmed the diversity and abundance of AMF, and different AMF species had different sensitivity to nitrogen application (Bhadalung et al., 2005). The accumulation of carbon and nitrogen may affect the adaptability of AMF to the soil environment, thus reducing AMF diversity (Ma et al., 2020). In this study, soil Shannon index was also negatively correlated with soil AP and AK. Studies have shown that the roots of plants absorb phosphorus from the soil in two ways. In soils with high phosphorus, the roots of plants can absorb phosphorus directly (Smith et al., 2015). In soils with low phosphorus, roots can become symbiotic with AMF and absorb phosphorus through hyphae (Johnson et al., 2015). Therefore, the diversity and abundance of AMF decrease with the increase of soil phosphorus content. Correlation analysis (Supplementary Table 2) showed that soil AMF Shannon index was negatively correlated with soil nutrient content. Soil nutrient content increased in the fertilization treatments, especially in the treatments of manure application, which may be the direct cause of the decrease in soil AMF diversity. Xu et al. (2016) have reported that soil pH was negatively correlated with AMF diversity. In our study, the soil pH had no significant effect on AMF diversity. This might be due to soil pH differences. Most of the soils tested in other studies were alkaline, whereas the soils in our study were more acidic (Jiang et al., 2020). In general, AMF taxa vary widely within their optimum pH ranges (Gai et al., 2006).

We also measured AMF diversity in maize roots to explore the differences. AMF diversity in maize roots was different from that in soil (Table 3). Shannon index of AMF in maize roots was the lowest in the N treatment. On the one hand, the Shannon index of AMF in maize roots was negatively correlated with soil NO_3_^–^-N (Supplementary Table 2); it might be due to that plant root exudates played an important role in the formation of the symbiotic system between AMF and plants, and NO_3_^–^-N could affect the composition of plant root exudates (Akiyama et al., 2005). On the other hand, the Shannon index of AMF in maize roots was positively correlated with soil pH; it might be due to that the decrease of pH was beneficial to the colonization of some AMF, which was more suitable to grow under acidic conditions. Generally, a low pH adversely affects the growth of AMF (Guo et al., 1996).

### Arbuscular Mycorrhizal Fungi Community Structure in Soil and Roots

A previous study showed that some AMF existing in rhizosphere soil do not appear in roots (Oliveira et al., 2009). In our study, a total of 12 genera of AMF were found in soil and root samples, of which three genera were not found in the maize root samples, i.e., *Archaeosporaceae*, *Acaulospora*, and *Innospora* (Figure 3). The species of AMF in roots were less than those in soil, which may be due to the high selectivity of host plants to AMF (Liu et al., 2019). AMF community structure in soil was influenced by soil physicochemical properties caused by fertilization. We found that the increase of soil nutrient content would harm the occurrence of AMF (Figure 3). Both in soil and maize roots, *Glomus* showed absolute dominance in the AMF community, indicating that *Glomus* was not sensitive to environmental changes caused by different fertilization compared with other AMF (Sasvári et al., 2011). One possible explanation for our observations is that *Glomus* gained an advantage at the expense of successful colonization of other AMF, whereas it could colonize new roots by hyphae or fragments, and it could survive and reproduce through mycelia, spores, or fragments (Daniell et al., 2001). The other possible reason is their high sporulation rate and strong symbiosis with plant roots; thus, *Glomus* is more resistant and resilient to ecological disturbances than other genera and may play an important role in performing ecological functions (Oehl et al., 2003). In this study, there were significant differences in the relative abundance of the AMF genus among different fertilization treatments (Figure 3). We found that the relative abundance of *Archaeosporaceae* and *Paraglomeraceae* in soils was very low, especially in the manure fertilizer treatments, which may be due to that *Archaeosporaceae* and *Paraglomeraceae* are widely distributed in natural ecosystems, and they are rare components of the AMF community in agroecological systems (Hijri et al., 2010). The relative abundance of *Claroideoglomus*, Paraglom*us*, *Scutellospora*, *Diversispora*, and *Dominikia* in soil decreased with the addition of manure, whereas *Septoglomus*, *Innospora*, and *Acaulospora* showed the opposite trend, which indicated that different AMF genera had different responses to nutrients. In this study, the relative abundance of *Claroideoglomus*, *Paraglomus*, and *Scutellospora* in roots decreased with the addition of manure, whereas that of *Septoglomus* showed an opposite trend (Figure 3B). These results showed that *Claroideoglomus*, *Paraglomus*, and *Scutellospora* were enriched in dystrophy conditions, whereas *Septoglomus* was more suitable to survive in eutrophic conditions. RDA confirmed our hypothesis (Figure 6).

Non-metric multidimensional scaling revealed that AMF community structure was significantly changed by different fertilization (Figure 4). AMF communities in soil were divided into two groups by whether applying manure. This may be because the application of manure can significantly improve the soil nutrient and soil organic matter content, and the increase of soil nutrients and organic matter provides abundant energy and nutrients for microbial growth, which may stimulate the growth of some AMF strains suitable for eutrophication. The AMF communities in roots were divided into two groups according to whether adding exogenous P fertilizer. This may be because the root exudates and nutritional status of P-deficient and non–P-deficient plants were significantly different; thus, the colonization of AMF in roots was also significantly different under these two conditions.

Redundancy analyse showed that AMF community structure in soil and roots was significantly affected by soil physicochemical properties and plant nutrients (Figure 6). AMF community structure in soil was affected by soil NH_4_^+^-N, NO_3_^–^-N, TN, SOC, TK, and AK, whereas AMF community structure in roots was affected by soil NH_4_^+^-N, NO_3_^–^-N, TN, SOC, TK, AK, AP, and plant phosphorus and potassium content. Previous studies have shown that long-term nitrogen and phosphorus applications have significant effects on AMF community composition (Zhang et al., 2020). With the increase of nitrogen supply, mycorrhiza in soils with high N content is inhibited compared to that in soils with low N content (Antoninka et al., 2011), which may be due to arbuscular formation in plant roots, depending on the exchange of exogenous C in exoplasts (Kiers et al., 2011). With the increase of nitrogen, the soluble C distributed in the carrier in plant roots decreased, inhibiting the diversity and abundant of AMF in rhizosphere soil and roots (Schwab et al., 1991). In this study, the long-term application of manure could affect the growth and composition of AMF in soil and roots, which may be due to its changes in the soil organic matter composition (Zhu et al., 2016). Studies have revealed that AMF can directly utilize simple organic matter (Govindarajulu et al., 2005); even some species with higher utilization efficiency of organic matter could obtain more energy sources for growth and reproduction and thus became the dominant species. The above reasons explained the correlation between AMF community and soil nitrogen content, organic carbon content in our study. Moreover, NO_3_^–^-N as one of the explanatory factors of AMF community structure in soil and roots may be due to an appropriate amount of NO_3_^–^-N, which could promote the symbiotic efficiency of mycorrhizal (Wang et al., 2020). The contents of soil AP and plant phosphorus content were the influencing factors of AMF community structure in roots, indicating that phosphorus played an important role in the colonization of AMF, and the presence/absence of P fertilizer has a significant effect on AMF communities (Cheng et al., 2013). In this study, potassium selectively stimulates the growth of AMF, which may be due to its compensation for Na-induced ionic imbalance (Hammer et al., 2011). So far, there are few studies on the effect of soil potassium on the AMF community, and further studies are needed.

## Conclusion

Long-term fertilization changed AMF community structure in soil and roots by changing soil physicochemical properties and plant nutrient content. AMF diversity in soil decreased with soil fertility, but AMF diversity in roots was influenced only by soil pH and NO_3_^–^-N. Long-term fertilization could improve the AMF spore density; especially, the long-term application of manure could greatly improve the AMF spore density by greatly improving soil fertility. The moderate content of soil AP was beneficial to the colonization of AMF. Soil NH_4_^+^-N, NO_3_^–^-N, TN, SOC, TK, and AK were the most important factors that influenced taxa of AMF in soil, whereas soil NH_4_^+^-N, NO_3_^–^-N, TN, SOC, TK, AK, AP, and plant phosphorus and potassium content were the most important factors influencing taxa of AMF in maize roots under long-term fertilization in brown soil.

## Data Availability Statement

The datasets presented in this study can be found in online repositories. The name of the repository and accession number can be found below: National Center for Biotechnology information (NCBI) BioProject, https://www.ncbi.nlm.nih.gov/bioproject/, PRJNA749871.

## Author Contributions

SZ, XH, and PL conceived, designed the study. JY, MI, JD, NA, and NL gave assisted in lab work and laboratory analyses. SZ and PL wrote the main manuscript text. XH contributed insightful discussions. All authors reviewed the manuscript.

## Conflict of Interest

The authors declare that the research was conducted in the absence of any commercial or financial relationships that could be construed as a potential conflict of interest.

## Publisher’s Note

All claims expressed in this article are solely those of the authors and do not necessarily represent those of their affiliated organizations, or those of the publisher, the editors and the reviewers. Any product that may be evaluated in this article, or claim that may be made by its manufacturer, is not guaranteed or endorsed by the publisher.
