# Supplementary material for: Responses of Arbuscular Mycorrhizal Fungi Diversity and Community to 41-Year Rotation Fertilization in Brown Soil Region of Northeast China
Source: Front Microbiol. 2021 Oct 11;12:742651. doi: 10.3389/fmicb.2021.742651 (PMC8542923; doi:10.3389/fmicb.2021.742651)
Supplement: Supplementary file 1 [file Data_Sheet_1.PDF]

Supplementary Material

Responses of arbuscular mycorrhizal fungi diversity and community to 41-year rotation fertilization in brown soil region of Northeast China

Shiyu Zhang[1,2,3], Peiyu Luo[1,2,3*], Jinfeng Yang[1,2,3], Muhammad Irfan[4], Jian Dai[1,2,3], Ning An[1,2,3], Na Li[1,2,3] and Xiaori Han[1,2,3*]

[1] College of Land and Environment, Shenyang Agricultural University, Shenyang, Liaoning, China

[2] National Engineering Laboratory for Efficient Utilization of Soil and Fertilizer Resources, Shenyang, Liaoning, China

[3] Scientific Observation and Experiment Station of Corn Nutrition and Fertilization in Northeast Agricultural and Rural Areas, Shenyang, Liaoning, China

[4] Department of Biotechnology, University of Sargodha, Sargodha, Pakistan

(A)

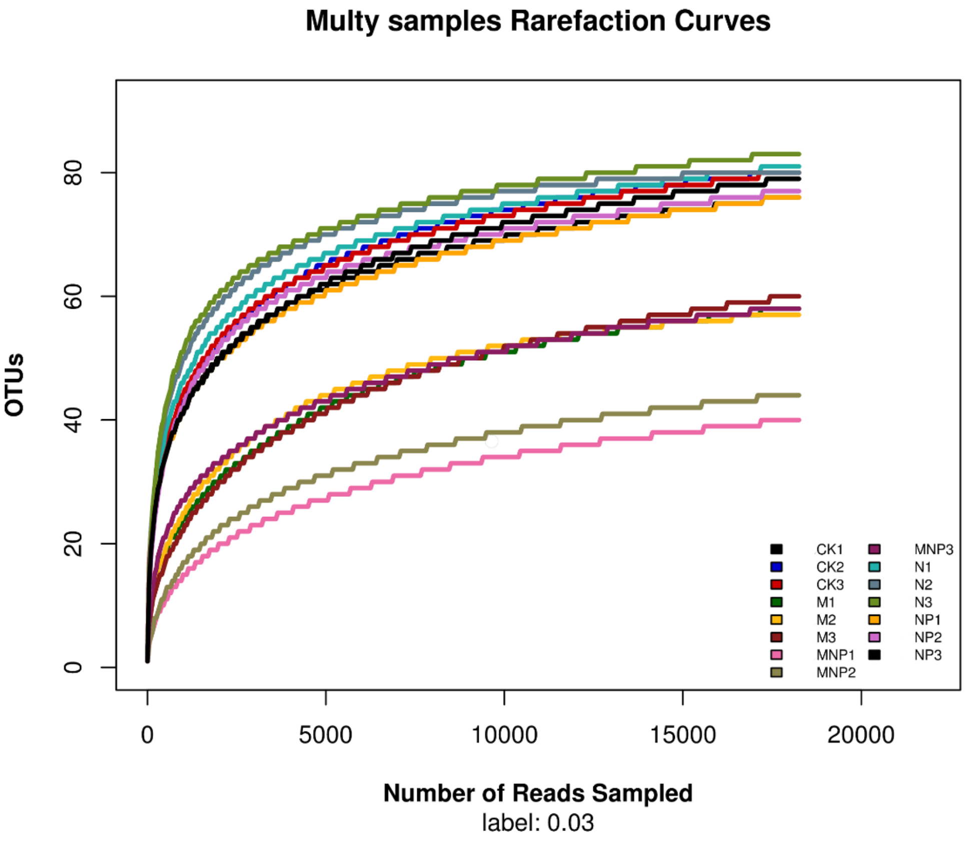

(B)

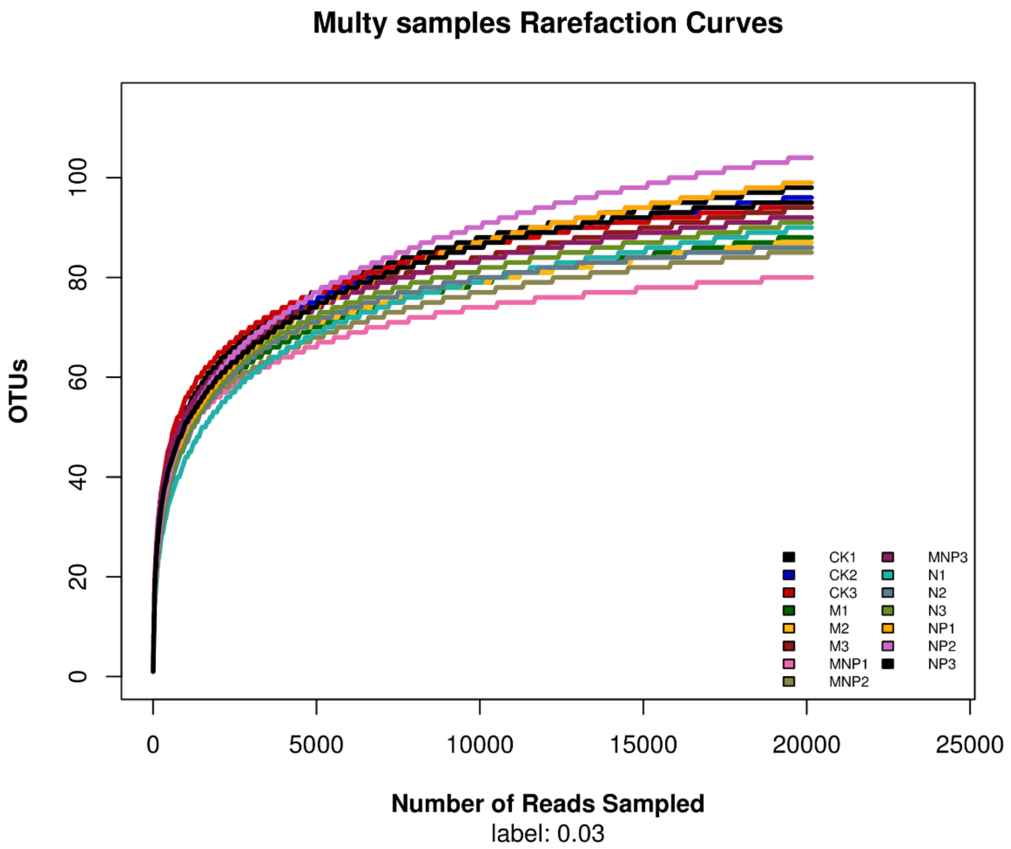

FIGURE S1 Rarefaction curves for the observed arbuscular mycorrhizal fungal (AMF) OTUs in soil **(A)** and maize roots **(B).**

Abbreviations: CK, no fertilizer; N, mineral nitrogen fertilizer; NP, mineral nitrogen and phosphate fertilizer; M, pig manure; MNP, pig manure, mineral nitrogen, and phosphate fertilizer.
