# Supplementary material for: Responses of Arbuscular Mycorrhizal Fungi Diversity and Community to 41-Year Rotation Fertilization in Brown Soil Region of Northeast China
Source: Front Microbiol. 2021 Oct 11;12:742651. doi: 10.3389/fmicb.2021.742651 (PMC8542923; doi:10.3389/fmicb.2021.742651)
Supplement: Supplementary file 2 [file Data_Sheet_2.docx]

Supplementary Material

Responses of arbuscular mycorrhizal fungi diversity and community to 41-year rotation fertilization in brown soil region of Northeast China

Shiyu Zhang^1,2,3^, Peiyu Luo^1,2,3*^, Jinfeng Yang^1,2,3^, Muhammad Irfan^4^, Jian Dai^1,2,3^, Ning An^1,2,3^, Na Li^1,2,3^ and Xiaori Han^1,2,3*^

^1^ College of Land and Environment, Shenyang Agricultural University, Shenyang, Liaoning, China

^2^ National Engineering Laboratory for Efficient Utilization of Soil and Fertilizer Resources, Shenyang, Liaoning, China

^3^ Scientific Observation and Experiment Station of Corn Nutrition and Fertilization in Northeast Agricultural and Rural Areas, Shenyang, Liaoning, China

^4^ Department of Biotechnology, University of Sargodha, Sargodha, Pakistan

| **Table S1** Application rates of fertilizer in maize of the treatments | | | | |
| --- | --- | --- | --- | --- |
| Treatment | Chemical fertilization rates (kg/hm^2^) | | | Pig manure(t/hm^2^) |
|  | N | P_2_O_5_ | K_2_O |  |
| CK | 0 | 0 | 0 | 0 |
| N | 120 | 0 | 0 | 0 |
| NP | 120 | 60 | 0 | 0 |
| M | 0 | 0 | 0 | 27 |
| MNP | 120 | 60 | 0 | 27 |
| Note: The long-term fertilization started since 1979, the rotation system was maize-maize-soybean, the rotation was repeated every three years  Abbreviations: CK, no fertilizer; N, mineral nitrogen fertilizer; NP, mineral nitrogen and phosphate fertilizer; M, pig manure; MNP, pig manure, mineral nitrogen and phosphate fertilizer. | | | | |

**Table S2** Correlation coefficients between the environmental factors and AMF-related parameters.

| Spearman | Spore density | AMF colonization | Shannon index of soil | Shannon index of roots |
| --- | --- | --- | --- | --- |
| NH_4_^+^-N | 0.867** | 0.576 | -0.964** | -0.030 |
| NO_3_^-^-N | 0.673* | 0.188 | -0.576 | -0.711* |
| AP | 0.884** | 0.604 | -0.994** | -0.009 |
| AK | 0.709* | -0.188 | -0.588 | 0.109 |
| SOC | 0.891** | 0.600 | -0.988** | -0.030 |
| TN | 0.881** | 0.575 | -0.966** | 0.015 |
| TP | 0.855** | 0.273 | -0.806** | 0.073 |
| TK | 0.952** | 0.127 | -0.806** | -0.219 |
| pH | 0.470 | 0.165 | -0.530 | 0.636* |

Note: * Significant at the 0.05 probability level, ** Significant at the 0.01 probability level.

Abbreviations: NH_4_^+^-N, ammonium nitrogen; NO_3_^−^-N, nitrate nitrogen; AP, available phosphorus; AK, available potassium; SOC, soil organic carbon; TN, total nitrogen; TP, total phosphorus; TK, total potassium.
